# How does context influence collaborative decision-making for health services planning, delivery and evaluation?

**DOI:** 10.1186/s12913-014-0545-x

**Published:** 2014-11-19

**Authors:** Anna R Gagliardi, Fiona Webster, Melissa C Brouwers, Nancy N Baxter, Antonio Finelli, Steven Gallinger

**Affiliations:** University Health Network, Toronto, Canada; University of Toronto, Toronto, Canada; McMaster University, Hamilton, Canada; St. Michael’s Hospital, Toronto, Canada

**Keywords:** Integrated knowledge translation, Health services research, Collaboration, Qualitative methods

## Abstract

**Background:**

Collaboration among researchers (clinician, non-clinician) and decision makers (managers, policy-makers, clinicians), referred to as integrated knowledge translation (IKT), enhances the relevance and use of research, leading to improved decision-making, policies, practice, and health care outcomes. However IKT is not widely practiced due to numerous challenges. This research explored how context influenced IKT as a means of identifying how IKT could be strengthened.

**Methods:**

This research investigated IKT in three health services programs for colon cancer screening, prostate cancer diagnosis, and the treatment of pancreatic cancer. Qualitative methods were used to explore contextual factors that influenced how IKT occurred, and its impact. Data were collected between September 1, 2012 and May 15, 2013 from relevant documents, observation of meetings, and interviews with researchers and decision-makers, analyzed using qualitative methods, and integrated.

**Results:**

Data were analyzed from 39 documents, observation of 6 meetings, and 36 interviews. IKT included interaction at meetings, joint undertaking of research, and development of guidelines. IKT was most prevalent in one program with leadership, clear goals, dedicated funding and other infrastructural resources, and an embedded researcher responsible for, and actively engaged in IKT. This program achieved a variety of social, research and health service outcomes despite mixed individual views about the value of IKT and the absence of a programmatic culture of IKT. Participants noted numerous challenges including lack of time and incentives, and recommendations to support IKT. A conceptual framework of factors that influence IKT and associated outcomes was generated, and can be used by others to plan or evaluate IKT.

**Conclusions:**

The findings can be applied by researchers, clinicians, managers or policy-makers to plan or improve collaborative decision-making for health services planning, delivery, evaluation or quality improvement. Further research is needed to explore whether these findings are widespread, and further understand how IKT can be optimized.

**Electronic supplementary material:**

The online version of this article (doi:10.1186/s12913-014-0545-x) contains supplementary material, which is available to authorized users.

## Background

For some time it has been known that interaction between knowledge producers and users is an influential means by which to generate practice-relevant knowledge and enable evidence-informed practice [1-3]. The imperative to improve evidence-informed decision making for health care planning, delivery, evaluation and improvement has prompted broader recognition of the need for such partnerships, and the conduct of research on how to optimize researcher-research user collaboration. Now more commonly referred to as integrated knowledge translation (IKT), this involves the development of a relationship between researchers and decision-makers (clinicians, managers, policy-makers, etc.) for the purpose of engaging in a mutually beneficial project or program of research [4]. It is distinguished from knowledge translation due to its emphasis on partnership or collaboration [5]. Decision-making research shows that complex problems require input from individuals with different but relevant expertise and perspectives to formulate, execute, and evaluate solutions [6]. Collaboration - like that promoted through IKT approaches - involves ongoing, dynamic interactions among researchers and decision-makers and represents an ideal means by which to address complex problems [7]. Proposed benefits of IKT include research questions that are more practice and policy relevant, and feasible to address; adaptable findings that are received by a primed and receptive audience; and an increase in mutual understanding of roles and values among researchers and decision-makers [4].

Empirical research has demonstrated concrete benefits of IKT. Interviews with researcher and National Health Services decision-maker partners of nine initiatives in the United Kingdom revealed that all achieved improved clinical care through a variety of IKT approaches [8]. An exploratory study of a university partnership with professionals in six Scottish health authorities revealed that communication improved, training was offered, and professionals reported enhanced skill and confidence, and a number of changes in their practice [9]. Early evaluation of an Australian obesity prevention network involving researchers at three universities and professionals at 78 community organizations found that the initiative resulted in the joint generation of best practice guidance and delivery of numerous professional development sessions [10]. Qualitative evaluation of an academic-primary healthcare collaborative initiative in the United Kingdom resulted in improved communication between agencies, identification of competency and knowledge deficits, and development and implementation of a tailored training program [11].

Despite this evidence of its positive impact on use of research, and improved health care planning, delivery and outcomes, we lack a thorough and shared understanding of its value and how to achieve it. In particular, IKT is challenged by the time and resources required for interaction; differential timelines between researchers and decision-makers; few professional and academic incentives tied to performance; and lack of interest, knowledge and skill to engage in IKT [4,6,12,13]. The need to foster IKT was recognized in both nursing and primary care sectors in Australia and the United Kingdom; among emergency medicine professionals from 16 countries; and by representatives of 33 research funding agencies in Canada, Australia, France, the Netherlands, Scandinavia, the United Kingdom, and United States [11,12,14,15]. A survey of health policy experts representing 30 European countries found that there were no explicit IKT mechanisms in most respondent countries other than a few examples where researchers were embedded in government research institutes or members of advisory committees [16]. The Canadian Academy of Health Sciences held a series of discussions with national leaders in 2004 and 2005 to address the lack of IKT for health services research and issued several recommendations, including the need for an audit of current and required capacity for IKT [13].

Existing research offers limited insight on approaches or strategies for operationalizing IKT. Reflection by those involved in a researcher-primary care partnership resulted in several recommendations to facilitate IKT including identifying partners with pre-established links to ease and expedite interaction; establishing clear expectations about role, scope and contribution to foster trust and avoid role confusion and misconceptions; fostering dialogue; and assessing progress to implement changes as needed [17]. A case study based on three health service delivery programs found that IKT was dynamic and highly influenced by the complex context within which decisions were being made including social and political norms [18]. Therefore, rather than imposing external or rigid IKT structures and processes, the investigators suggested that IKT could be optimized by first examining naturalistic interaction that takes place in a given program or organization to identify how IKT could be supported. They proposed that, by considering contextual influencing factors, both barriers and facilitators, existing mechanisms could be enhanced, and IKT may be more likely to be accepted and applied.

Context is widely recognized as a multi-dimensional factor that must be accommodated when tailoring, adapting, implementing, scaling-up or spreading programs or interventions to optimize impact and enhance sustainability [16,19]. Context has been referred to as anything that cannot be described as an intervention or its outcome [20]. A recent review and amalgamation of determinants of practice identified 57 unique factors that were grouped in seven domains: guideline/innovation, individual health professional, patient, professional interaction, incentives and resources, capacity for organizational change, and social, political and legal factors [21]. The Promoting Action on Research Implementation in Health Services (PARIHS) framework captures several of these factors, and has been used as the basis for planning or evaluating interventions, and the factors that influence their success [22]. The PARIHS framework suggests that the nature of evidence, the context in which it is applied, and facilitation of the implementation or adoption process together influence research use. Research in clinical settings highlighted the importance of context relative to evidence and facilitation, and that context includes micro (individual) and meso (organizational) level factors [23,24]. According to PARIHS context is comprised of an organizational culture that is receptive to change, leadership that supports the involvement of individual staff, and evaluation and feedback mechanisms [22-24].

Empirical evidence shows that IKT improves healthcare planning, delivery, evaluation and quality improvement but due to a variety of challenges is not widely practiced. Further research is needed to reveal how IKT can be promoted and supported. Context refers to a broad array of factors that can influence the practice or success of interventions, but the contextual factors that influence IKT have not been examined. The purpose of this research has therefore been to identify contextual factors influencing IKT in different healthcare planning or improvement programs and, based on the findings, generate a conceptual framework by which others could plan, promote, strengthen or evaluate IKT.

## Methods

### Approach

This research took place in the setting of Ontario, Canada and employed a qualitative approach to explore the use and impact of IKT, including how researchers interacted with decision-makers and how their research or knowledge about research was used by decision-makers. This was examined in three health services programs that sought to improve underuse of an organized colon cancer screening program, overuse of prostate cancer screening, and high mortality of pancreatic cancer. Qualitative research is useful when there is a need to develop a rich and thorough appreciation of the contextual factors that influence views and behaviour when there has been little prior research [25]. Rigour was optimized by integrating data (“triangulation) collected in variety of ways (interviews, observation, documents) from different sources (decision-makers, researchers); sampling participants with various characteristics that could influence their views; fully analyzing and interpreting data including deviant cases; checking findings with participants; demonstrating responses from an array of participants by including an anonymous identification code with exemplary quotes; and comparison of independently-derived thematic coding across two individuals [26]. Rigour was further ensured by complying with Relevance, Appropriateness, Transparency and Soundness (RATS) principles for the reporting of qualitative research [27] as the citation in Additional file 1. Ethics approval for this study was acquired from the University Health Network which required that participants provide written informed consent prior to being interviewed. Those observed at meetings provided unanimous oral consent before the start of the meeting. In this study IKT was defined as interaction among researchers and decision-makers for program planning or evaluation, or research.

### Theoretical framework

There is no single or comprehensive theory or model that encompasses the influencing contextual factors, processes and outcomes of IKT so the goal of this research was to generate, rather than prove existing theory. Naturalistic mechanisms of IKT in the involved programs, associated outcomes and, in particular, contextual factors that emerged from the data were compiled in a conceptual framework of contextual factors that influence IKT practice and impact. To analyze data and identify unique findings, emerging contextual factors were examined according to the components of the PARIHS framework including culture (normative views and behaviour), leadership (visibly involved in activities and actively engage staff), and evaluation (feedback is provided to staff on performance and improvement) [22].

### Sampling and recruitment

Three programs were chosen as they had been identified as priorities by the provincial cancer agency, and were thought to differ in a number of ways that could influence IKT including type and prevalence of cancer, health service issue, type of stakeholders, and quantity and quality of evidence underlying the health services issue. For each program, a lead key informant was identified. They suggested and brokered links with individuals for interviews, some of whom recommended additional individuals for interviews. Lead key informants also identified relevant meetings for observation, and documents for content analysis. Purposive sampling was used to recruit interview participants. The goal was to interview five researchers and five decision-makers in each of the three programs for a minimum of 30 interviews. Researchers included clinician and non-clinician/PhD researchers. Decision makers included policy-makers, managers, or clinicians who were affiliated with the chosen programs. Detailed information from representative informants, rather than a large number of participants is needed in qualitative research. Recruitment was concurrent with data collection and analysis, and continued until saturation was achieved, meaning no further unique themes emerged from successive interviews as determined by discussion between two independent reviewers [25].

### Data collection and analysis

Data was collected between September 1, 2012 and May 15, 2013. Document analysis: Content analysis is used to describe phenomena in written, verbal or visual communication [28]. The content of strategic plans, reports, articles, meeting minutes, web sites and other documents referred to, or provided by participants were examined using directed content analysis techniques [29]. This means that the explicit content was coded for direct or indirect instances of collaboration. Data were extracted by a trained research assistant from relevant documents using a structured guide that included document name, location, type, purpose and date of publication, authors and roles, and any direct or indirect evidence of researcher-decision-maker collaboration or influence of researchers on decisions. The principal investigator re-examined all documents to confirm collected data. Field Notes: Field work included the observation of meetings to identify whether and how researchers interacted with decision-makers, and were involved in influencing decisions. Coordinators of relevant committees were contacted to request access to meetings. Data were collected by a trained research assistant who attended all meetings and used a structured guide to document meeting purpose, location, duration, number and role of participants, nature of interaction, and researcher involvement in or influence on decisions. Field notes were summarized to describe observed interaction or mention of interaction between researchers and decision-makers during meetings. Interviews: Grounded theory was used to collect and analyze interviews data [30]. This means that ideas emerged inductively from the data and were then compared with existing theory, rather than using theory to structure data collection which can restrict exploration and miss relevant themes. Interview candidates were contacted by email with an invitation and consent form. Semi-structured interviews were conducted by a trained research assistant via telephone, audio-recorded and transcribed verbatim. Participants were asked four questions – to describe their understanding of IKT, IKT activities in which they were involved, challenges of IKT, and recommendations for how it could be promoted and enabled. Mean length of interviews was 29.47 minutes (median 29.10, range 16.01 to 57.11). Interview transcripts were analyzed iteratively using constant comparison to identify, code and organize themes [31,32]. Initially, open coding was performed to get a general feel for the content of the data, then more selective coding was performed after core concepts began to emerge. Three members of the research team independently read and coded the first three interview transcripts, then met to compare their analyses and developed a codebook that guided subsequent analysis of remaining transcripts. Data Integration: Data from interviews, field work and document analysis were integrated using relational analysis [33]. Key findings from each mechanism of data collection were listed (program description by lead key informants, document analysis, observation, interviews). All unique components were categorized as IKT processes, outcomes or contextual factors which were further organized as individual or organizational level contextual factors, and then as sub-categories. The integrated findings were summarized both textually and visually. Contextual factors influencing IKT that emerged from the study were compared with the components of the PARIHS framework [22-24]. A summary of the analysis was prepared, shared with the study team (“member checking”), and refined based on their feedback.

## Results

### Overview of clinical programs

The three clinical programs are described in Additional file 2, and key characteristics are highlighted here. The colorectal cancer screening program was described by the lead key informant as “established”. It was conceptualized from 1999 to 2002, a pilot program was completed in 2006, and a population-based program was implemented in 2008. Its explicit goals are to reduce deaths from colorectal cancer by inviting and reminding individuals for screening, tracking individuals throughout screening and diagnosis, and providing support for screening to health care providers. Evaluation reports released in 2010 and 2012 both highlighted increased participation in screening, though rates were lower than targeted. The program is funded by the provincial Ministry of Health and cancer agency, operates a secretariat with leadership and staff at the cancer agency. Its activities are guided by several different working groups or committees that include health professionals and researchers from across the province. The prostate cancer program was described by the lead key informant as in the “beginning stages”. It was not a formally recognized program with clearly stated goals or infrastructure. Activities including several meetings during which leaders from the cancer agency gathered health professionals and researchers from across the province to identify quality improvement issues and generate recommendations for ongoing activity. Similar to the prostate cancer program, the pancreatic cancer program did not have formal status with particular goals or infrastructure. While considerable activity had taken place as early as 2006 and over several subsequent year to evaluate quality of surgical care and associated outcomes, and issue recommendations for hospitals delivering such services. The program was in a period of dormancy as research priorities were shifting from treatment to identifying the molecular mechanism of disease which might lead to prevention or early identification of disease. It was described by the lead key informant as an “upcoming priority”.

### Document analysis – interaction through meetings, research, guideline development

Documents examined (27 colorectal, 8 prostate, 4 pancreatic) included strategic plans or proposals, meeting minutes, meeting or workshop summary reports, program evaluations, clinical practice guidelines and published research papers. There was no explicit description of interaction between researchers and decision-makers. However, physicians and physician-researchers were named as co-authors on a few documents relevant to prostate cancer diagnosis and pancreatic cancer treatment; and managers, physicians, physician researchers and PhD researchers were named as co-authors on several documents relevant to colorectal cancer screening. This implies that IKT took place, more so for the colorectal program, in the form of interaction at formal meetings, collaboration for research, and joint guideline development. Details of timing, frequency, level of interaction and impact (other than publications or reports) were lacking.

### Meeting observation – researchers supported and influenced decision-making

No meetings were observed relevant to prostate or pancreatic cancer because none were scheduled during the study period. Six meetings relevant to the colorectal context were observed. Physician researchers who were embedded as staff in, or affiliated with or appointed to programs or committees appeared to be involved in decision-making and in guiding decision-making in a variety of ways. They did so by having summarized available evidence for review at the meeting, describing and commenting on the quantity and quality of the evidence, interpreting the evidence and whether and how it was relevant or could be applied, and raising issues or implications, or offering suggestions to guide further decision-making. They also offered examples based on their own clinical experience, recommendations from published guidelines, and known risk factors from published research. While the impact of these efforts could not be discerned, formal meetings at which researchers were present appeared to offer opportunities for IKT to occur.

### Interviews – numerous contextual factors challenge IKT

Of 77 individuals invited to participate in interviews, 36 consented and were interviewed (Table 1), nine declined, and 32 did not respond. Key findings are described here, and exemplar quotes from participants supporting these themes are summarized in Table 2. Views expressed by interview participants were similar across the three programs. Most participants understood that the purpose of IKT was to promote the use of research in practice. However, they described IKT in terms of traditional dissemination involving one-way transfer of knowledge rather than an interactive or collaborative process. Most participants thought that IKT resulted in mutual learning and professional benefit for researchers and decision-makers, and greater understanding of differing perspectives. IKT was thought to enhance the efficiency of conducting research, and the quality and relevance of research leading to greater and accelerated use of research that improved the decision-making process, and subsequent policy, practice and health care outcomes. Physician researchers who were embedded in or formally affiliated with a program were more likely to report interaction with decision-makers compared with external researchers without any form of affiliation. Most interaction occurred between physician researchers or managers and physician end-users. When it occurred, IKT largely took place at regularly scheduled committee meetings. Managers expressed little interest in, or uncertainty about the purpose of engaging researchers. If this occurred, it was most likely at the end of a project. Instead they relied on published research, valuing currency and explicitly stated relevance in such reports.

**Table 1:** **Professional role of study participants**

**Program**	**Researchers**		**Decision-makers**		**Total**
	**Clinician**	**Non-clinician**	**Clinician**	**Manager**	
Colorectal	4	3	2	3	12
Prostate	7	1	-	3	11
Pancreatic	5	1	-	-	6
General Context*	3	-	-	4	7
Total	19	5	2	10	36

*Participants whose roles were not restricted to a particular program.

**Table 2:** **Interview themes**

**Theme**	**Sub-themes**	**Exemplar quote**
Awareness or knowledge of IKT	Unaware or unclear	It’s not a term that I commonly use [012CO/R]
	Equated with dissemination	It’s the process whereby information that has been generated scientifically is transmitted to others who have interest in the knowledge [017CO/RU]
	Understanding of collaborative nature	A relationship between knowledge users and producers that can be on-going [010CO/U]
	Researchers initiated/used IKT	I have to make sure that what I’m finding gets out there [002CO/R]
	Need for tailoring to context/audience	Depending on who your audience is, you try and construct the activity to the audience [007CO/U]
Benefit of IKT	Improved the relevance and quality of research	Without that dialogue the likelihood of producing something that’s relevant in the healthcare context that you wish to impact on is lower [004CO/R]
	Inform new research questions	Stakeholders can also inform the researchers in what are research priorities etc. [011GE/RU]
	Increase efficiency of research	Ultimately save time, money and other resources [032PR/R]
	Facilitated learning relevant to professional tasks	There’s mutual learning and assistance to help the various participants move ahead in whatever their agenda is [022PR/RU]
	Enhanced understanding of different perspectives	Useful for researchers to understand what the research priorities are from the perspective of the policy- makers [014GE/R]
	Promote use of research	You’re creating a ready-made receptor for the knowledge along the way [022PR/RU]
	Improved decision-making, policy, practice and outcomes	Bring knowledge to practice in order to improve outcomes [030PR/R]
	Accelerates production and use of research	The best way to accelerate the process of integration of best evidence in practice, policy, etc. [014GE/R]
Engagement in IKT	Embedded researchers interacted with managers	Because I’m embedded…that allows that exchange of knowledge to happen [004CO/R]
	Focus on interaction with clinicians	Much of my work in terms of presentations has been to clinical people [030PR/R]
	Managers unclear about benefit	I don’t know where there would be a benefit of direct interaction with researchers [020GE/U]
	Researchers engaged in program evaluation	The only time that we have [involved researchers] is after the fact [006GE/U]
	Managers prefer learning about research through publications	I really like systematic reviews of evidence so that I don’t have to sift through all of the stuff that’s out there [009CO/U]
	IKT took place in regularly scheduled meetings	It’s generally a meeting over video-conference or a face-to-face meeting [018PR/U]
Challenges and enablers of IKT	Willingness to collaborate	They’re responsive and willing to learn, and collaborate with us [008CO/R]
	Innate ability to collaborate	Some people are good at committees, some are not. Stipend or formal position won’t change that [003PA/R]
	Motivation and incentives	Researchers often are involved in research as a pure academic pursuit so they don’t think through the need to share that information quickly with policy-makers. They’re focused on peer-reviewed publication as their reward [019PR/U]
	Need for formal linkages	You have to be linked to a program or service to really know what’s going on [002CO/R]
	Responsibility unclear	It’s me who is initiating the request [to interact with policy makers] [008CO/R]
	Awareness of, or access to opportunities	We don’t have the means to know about all the other forums for exchange that are going on and often they’re invitation only for people that are clinicians [029PR/U]
	Resistance to change	Users are not necessarily so open to embracing change [023PR/RU]
	Unintended transformation or use of research findings	It transforms the product so much that you’ve lost what was the principle research finding [004CO/R]
	Unsynchronized timelines	It’s just synchronizing everyone towards the same goal at the same time [028PR/RU]
	Organizational endorsement	There’s got to be buy-in from the institution [033PR/R]
	Leadership advocacy	The manager is the interface [013GE/RU]
	Mentors and champions	Having a local champion has been helpful [025PA/RU]
	Time required	The biggest challenge would just simply that everyone’s very, very busy [003PA/R]
	Resources and incentives	It’s impossible to get very busy people to do this kind of work without (compensation) [002CO/R]
	Difficult to evaluate and show impact	It’s difficult to measure and also difficult to find an impact [017GE/RU]

IKT was challenged by multiple factors that included interest in, and skills for collaboration; organizational recognition of, and support for IKT through leadership, mentors, champions and brokers; a mismatch in timing and goals between researchers and decision-makers; limited funding, resources and time for IKT; lack of coordination or integration across programs even within a single organization; and resistance to change. Decision-makers thought that researchers did not reach out to them, while researchers said they experienced difficulty accessing decision-makers and opportunities for interaction. Participants were asked to suggest strategies for overcoming challenges, and promoting and enabling IKT (Table 3). They said that IKT could be strengthened by recognizing it as an explicit organizational priority along with IKT-specific strategic plans, designated leaders, resources and regular forums to support it, providing researchers with formal affiliations and opportunities for interaction, and enabling it through education, brokers and an inventory of research. They thought that greater awareness of the impact of IKT might promote its use. A few contradictions were identified. For example, there was disagreement on whether more meetings to enable IKT were desirable, and a few participants recommended the use of technology-enabled communication. While some participants said that researchers should be embedded in every standing committee, others said that evidence could instead be acquired from published literature, or that researchers were biased, or that they may have a role only at the end of a program to evaluate its impact.

**Table 3:** **Recommendations to support IKT**

**Challenge expressed by participants**	**Solutions recommended or inferred**
Awareness of/knowledge about IKT	• Organizational culture
	• Champions
	• Training
Funding, resources, time for IKT	• Organizational culture
	• IKT-specific strategic plan
	• Demonstrate impact that can be derived
Attitude about IKT	• Organizational culture
	• Champions
	• Demonstrate impact that can be derived
Willingness to collaborate, resistance to change	• Leadership
	• Champions
	• Professional and academic incentives
	• Demonstrate impact that can be derived
Skill for collaboration	• Training
	• Mentorship
Access to opportunities	• IKT-specific strategic plan
	• Knowledge brokers/facilitators
	• Formal program affiliations for researchers
	• Inventory of initiatives/research
	• Use of technology-enabled communication tools
Mismatch timing/goals between researchers and decision-makers	• Training
	• Knowledge brokers/facilitators
Coordination/integration across programs	• IKT-specific strategic plan
	• Knowledge brokers/facilitators
Variety of forums for IKT	• IKT-specific strategic plan
	• Use of technology-enabled communication
Responsibility for initiating IKT	• Organizational culture
	• Training
	• Mentorship
	• Knowledge brokers/facilitators
	• IKT -specific strategic plan
	• Professional and academic incentives

### Summary of integrated findings

**Figure 1:**
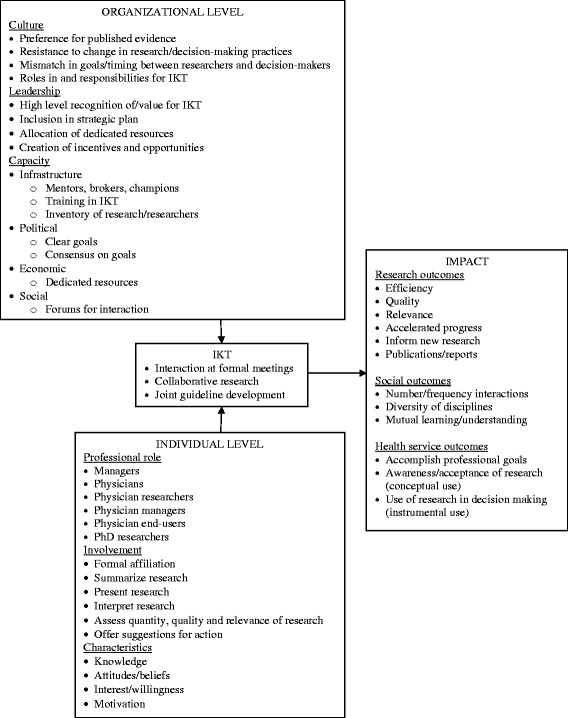
**Conceptual framework of contextual factors influencing IKT practice and impact.**

Contextual factors that influenced the type, prevalence and impact of IKT are summarized in Figure 1. Document analysis identified that naturalistic or typical means of IKT across the three programs included collaborative research, joint guideline development and, most often, interaction at formal or regularly scheduled meetings. Observation of meetings identified that researchers actively contributed to decision making in a variety of ways including summarizing, presenting, interpreting and commenting on the quantity, quality and relevance of evidence, and whether and how it could inform decisions. More documents and meetings were available for the colorectal program compared with either the prostate or pancreatic program. This was largely due to key differences in program characteristics. The colorectal program was funded by the government so there were dedicated resources to operationalize and evaluate the program. Infrastructure was in place including leadership and administrative support from a centralized office. The program also appeared to have a clear, specific and agreed upon focus. Various stakeholders had roles and were engaged in standing committees, including one researcher committee, one clinician committee, and several interdisciplinary committees which featured greater professional diversity compared with the prostate and pancreatic programs. Thus there were ongoing forums for interaction. In particular, the colorectal program featured an embedded scientist who participated in most of the committees and was specifically hired to contribute to program planning and evaluation. Given the level of support and activity in the colorectal program, the impact of IKT was apparent as several types of outcomes. With respect to social outcomes, the number and frequency of interactions and diversity of engaged disciplines appeared greater than the other programs as did research outcomes including number of products and publications. With respect to health service outcomes, evidence was both used and generated in program planning, delivery and evaluation, though desired clinical outcomes such as increased use of screening were not achieved. Interviews largely confirmed that leadership, infrastructure, political support in the form of clear, agreed-upon goals, dedicated resources, and opportunities for social interaction all contributed to greater IKT and associated impact in the colorectal program. However, in all three programs some tensions were evident that represent IKT challenges. These included individual willingness to take part in IKT, lack of institutional incentives or recognition for IKT, ambiguous responsibility for IKT, and cultural factors that created mismatches in decision maker and researcher goals, and a reliance on more traditional forms of sharing or acquiring evidence. Thus it is notable that the more established, IKT-active program achieved a variety of outcomes based on leadership, clear goals, dedicated funding and other infrastructural resources, and an embedded researcher responsible for, and actively engaged in IKT, despite mixed individual views about the value of IKT and the absence of a programmatic culture of IKT.

### Conceptual analysis of integrated findings

Study findings confirmed several components of the PARIHS framework. The PARIHS framework suggests that research use is influenced by context, where context is defined as a culture receptive to change, leadership support, and feedback to staff. In this research both culture and leadership appeared to be associated with IKT and associated outcomes, though feedback to staff was not evident as either an influencing factor, IKT process or an outcome. Cultural factors that were relevant to this research included reliance on published evidence; resistance to change on the part of both researchers and decision-makers; mismatch between the goals and timing of researchers and decision-makers; and lack of clarity around IKT roles and responsibilities. While leadership was apparent in the colorectal program, in part accounting for IKT activity, participants of this and other programs recommended other forms of leadership or leadership activities including high level recognition for IKT as an organizational priority described in strategic plans, incentives for IKT, and greater opportunities for interaction. Study findings revealed several additional contextual factors that influenced IKT beyond the components of PARIHS. These included organizational capacity, which could be further categorized according to socio-political (clear goals, consensus on goals), economic (dedicated funding, resources and infrastructure such as space, administrative support) and social (formal and informal interaction, affiliated or embedded roles, diversity of engaged disciplines) factors that either enabled or challenged IKT. Findings also confirm the relevance of contextual factors at the individual (knowledge, beliefs, motivation) and organizational (culture, leadership, capacity) levels. It is notable that in this study leadership and capacity (infrastructure, funding, clear goals), in the absence of a strong IKT culture, were sufficient to achieve tangible outcomes including a variety of social, research and health service outcomes, which also elaborated the PARIHS description of outcomes beyond the general term of research use.

## Discussion

This study was conducted to examine how context influences IKT and, in so doing, generate a conceptual framework of factors that influence IKT practice and outcomes that could be used to strengthen IKT and guide ongoing research. Regular meetings of researchers, decision-makers and end-users were the most common forum for IKT. Meetings were most prevalent in the program that featured some contextual factors identified by PARIHS [22-24], in particular leadership and capacity (infrastructure, dedicated funding, clear goals) and several additional contextual factors and associated outcomes identified by this research, and which achieved a variety of social, research and health service outcomes. The additional contextual factors unique to this research have been captured by sociological theories including social, political, and economic factors that may influence individual beliefs and practices, or programmatic support for IKT [34]. The outcomes achieved by the more established and IKT-active program included use of research considered as conceptual (awareness, acceptance) and instrumental (decision-making) [35]. Findings were captured in a conceptual framework of factors that influence IKT practice and outcomes. Recommendations were issued for a variety of strategies that could support and optimize IKT. Recommendations were similar to factors that enabled collaboration in literature on management networks, interprofessional health services research, teamwork, knowledge translation, communities of practice, and quality improvement collaboratives [36].

The interpretation and application of study findings may be limited by several issues. Few documents and meetings were available for two of three programs, however, that data was retained to compare the lack of development of those programs and in associated IKT given the limited nature of certain contextual factors such as program funding, leadership, infrastructure and joint goals. We also may not have interviewed all relevant stakeholders associated with each program, partly due to non-response. However we did sample for interviews according to program and professional role within each program so findings likely captured a variety of views that may have been influenced by these factors, and interview data was supplemented with information from content analysis of documents and meeting observation. The chosen programs may not have revealed a variety of IKT activity or possible factors influencing IKT given that two contexts were not well established or active. Further research of a similar nature but in additional programs would extend these findings and further examine the influence of context on the prevalence, nature and outcomes of IKT.

Study findings confirm those of a case study that evaluated three health service delivery programs which found that IKT was highly influenced by the complex context within which decisions were made including social and political issues [18]. It is recommended that these contextual factors be considered when tailoring or implementing programs or interventions [16]. Other research that evaluated the influence of context focused on the implementation or adoption of clinical practices in primary, acute and long-term care settings [37,38]. The conceptual framework generated here provides organizations with a means of planning or evaluating contextual issues specific to the use and impact of IKT. Existing research offers limited insight on the approaches or strategies by which to operationalize IKT. Suggestions generated by other researchers included identifying partners with pre-established links to ease and expedite interaction; establishing clear expectations about role, scope and contribution to foster trust and avoid role confusion and misconceptions; fostering dialogue; and assessing progress to implement changes as needed [17]. These findings add to that body of knowledge by offering a detailed framework of individual and organizational contextual factors to consider.

A key role appeared to be that of an embedded scientist. This was identified as one of the few mechanisms for enabling IKT in a survey of health policy experts representing 30 European countries [16]. This individual was an active participant in planning committees, generated and contributed to research, and offered information and insight that influenced decisions about program planning and evaluation. Further research should explore the role of embedded scientists to elaborate on the scope of such roles, how they can be supported and their impact. While this research did identify contextual factors that influenced IKT along with challenges and suggested enablers, it identified few naturalistic IKT activities upon which to build, an approach theorized to result in greater acceptance and sustained use of IKT [18]. When it occurred, most interaction took place at committee meetings, and through joint development of guidelines and collaborative research. Team meetings and teleconferences were also the main IKT activity in an international network of over 60 researchers, trainees and decision-maker partners from Canada, the United States, the United Kingdom, Asia, Europe and Australia after its first three years of operation [39]. Views expressed by middle and high level managers suggest they prefer published research over interaction with researchers, and non-embedded researchers noted that it was difficult to access opportunities for collaboration. Overall, most individuals had a limited understanding of IKT and how it could be achieved, and disagreed on whether more meetings were desirable. Other research has identified these and other challenges, including a lack of incentives for undertaking IKT [4,6,12,13]. That was certainly evident here. Research is needed to further understand this tension between lack of time and incentives for interaction, and the need for interaction to support IKT. Such research may reveal options other than meetings by which to achieve IKT.

Several studies have identified tangible improvements in health services or clinical outcomes as a result of IKT [8-11]. Several positive outcomes were achieved in the participating programs but not health service outcomes. While that may be due to the limited infrastructure for, and challenges of IKT identified in the contexts studied here, longitudinal evaluation may be needed since such impact may not be immediate. A study that examined self-reported research use by pediatric nurses found that cultural norms and formal interaction were negatively associated with conceptual research use (cognitive awareness and acceptance), which is thought to be a precursor to instrumental research use (actual application in decision-making or behavior) [35]. This emphasizes the need to longitudinally examine a variety of outcomes associated with IKT including cognitive impact. Kothari et al. generated indicators reflecting the capacity for, and impact of collaboration by interviewing researchers and policy decision-makers affiliated with a ten-year Ministry of Health and Long Term Care initiative [40]. The indicators included measures of partnership and measures reflecting the impact of partnership that differed early in the relationship and longitudinally after partnerships matured.

## Conclusion

Regular meetings of researchers, decision-makers and end-users were the most common forum for IKT across all programs. Meetings were most prevalent in the program with leadership, clear goals, dedicated funding and other infrastructural resources, and an embedded researcher responsible for, and actively engaged in IKT. This program achieved a variety of social, research and health service outcomes despite mixed individual views about the value of IKT and the absence of a programmatic culture of IKT. Together with challenges and enablers identified by participants, this study generated a conceptual framework of factors that influence IKT practice and outcomes which can be used by others to plan or evaluate IKT. Further research is needed to explore whether these findings are widespread, and understand how IKT can be optimized. However, these findings can be applied by researchers, clinicians, managers or policy-makers to plan or improve collaborative decision-making for health services planning, delivery, evaluation or quality improvement.

## Additional files

Additional file 1:
**Relevance, Appropriateness, Transparency and Soundness (RATS) principles for the reporting of qualitative research.**
Additional file 2:
**Overview of Clinical Contexts.**
