# Supplementary material for: How does context influence collaborative decision-making for health services planning, delivery and evaluation?
Source: BMC Health Serv Res. 2014 Nov 19;14:545. doi: 10.1186/s12913-014-0545-x (PMC4239386; doi:10.1186/s12913-014-0545-x)
Supplement: Additional file 1: — Relevance, Appropriateness, Transparency and Soundness (RATS) principles for the reporting of qualitative research. [file 12913_2014_545_MOESM1_ESM.doc]

**Relevance, Appropriateness, Transparency and Soundness (RATS)
principles for the reporting of qualitative research**

Clark JP: How to peer review a qualitative manuscript. In Peer Review in Health Sciences. Second edition. Edited by Godlee F, Jefferson T. London: BMJ Books; 2003:219-235

| **RATS Domains and Items** | **Page in manuscript** |
| --- | --- |
| **R – relevance of study question** |  |
| Research question explicitly stated | 7-8 |
| Research question justified and linked to the existing knowledge base (empirical research, theory, policy) | 4-7 |
| **A – appropriateness of qualitative method** |  |
| Study design described and justified i.e., why was a particular method (e.g., interviews) chosen | 8 |
| **T – transparency of procedures** |  |
| Criteria for selecting the study sample justified and explained | 9-10 |
| Details of how recruitment was conducted and by whom | 9-10 |
| Details of who chose not to participate and why | 14 |
| Data collection method outlined | 10-11 |
| Study group and setting clearly described | 12-13, supplement 1 |
| End of data collection justified and described | 10 |
| Do the researchers occupy dual roles (clinician and researcher)? Are the ethics of this discussed | n/a |
| Ethics approval cited | 8 |
| Informed consent process explicitly and clearly detailed | 8 |
| **S – soundness of interpretive approach** |  |
| Analytic approach described in depth and justified: Description of how themes were derived from the data (inductive or deductive); Evidence of alternative explanations being sought; Analysis and presentation of negative or deviant cases | 11-12 |
| Description of the basis on which quotes were chosen: Semi-quantification when appropriate; Illumination of context and/or meaning, richly detailed | 11 |
| Method of reliability check described and justified: e.g., was an audit trail, triangulation, or member checking employed? Did an independent analyst review data and contest themes? How were disagreements resolved? | 8,10,12 |
| Strengths and limitations explicitly described and discussed | 19 |
| Detail of methods or additional quotes contained in appendix | Supplement 1 |
