# Supplementary material for: How does context influence collaborative decision-making for health services planning, delivery and evaluation?
Source: BMC Health Serv Res. 2014 Nov 19;14:545. doi: 10.1186/s12913-014-0545-x (PMC4239386; doi:10.1186/s12913-014-0545-x)
Supplement: Additional file 2: — Overview of Clinical Contexts. [file 12913_2014_545_MOESM2_ESM.doc]

**Overview of Clinical Contexts**

A general understanding of the three clinical programs was established before full launch of the study with information gathered from lead key informants and from documents they recommended.

Colorectal Cancer Screening

Colorectal cancer (CRC) has a high chance of being cured if detected early through screening. After recommendations by two CRC expert panels in 1999 and 2002[[1]](#footnote-2), a proposal was put forth to develop and implement a fecal occult blood testing (FOBT)-based CRC screening program in Ontario for average risk individuals between the ages of 50 and 75. A pilot project was completed in 2006 which demonstrated the effectiveness of FOBT promotion and recruitment[[2]](#footnote-3), as well as a report released in 2007, defining usability, laboratory and performance standards of FOBT in CRC screening[[3]](#footnote-4). Finally in April of 2008, Colon Cancer Check (CCC), Canada’s first population-based province-wide CRC screening program, was publicly launched. The inaugural CCC program evaluation was released in 2010 and highlights include a doubling of FOBT participation rate from 14.8% in 2003-2004 to 29.7% in 2007-2008, with the increase seen in all geographic regions of Ontario[[4]](#footnote-5). Despite promising increases, the reported rates were below Ontario Cancer Plan targets. The second CCC program report released in 2012 revealed decreased FOBT participation of 27% in 2009-2010, also falling short of the 2010 targeted rate of 36%. In 2011, it was reported that a new form of CRC screening, fecal immunochemical testing (FIT), had higher screening participation rates and greater sensitivity than FOBT[[5]](#footnote-6). This launched a pilot project to determine how to optimally implement FIT in CCC. Results are expected in 2013 and will be used by Cancer Care Ontario (CCO)/Ministry Health and Long-Term Care to prepare for switching to FIT as the primary mode of CRC screening.

Prostate Cancer Diagnosis

Prostate cancer is the most frequently diagnosed cancer in Ontario, but can often be cured or managed successfully. A blood test for high levels of prostate specific antigen (PSA) based on age or increase over the years, may lead to imaging and biopsy studies before diagnosis. However, PSA test screening in asymptomatic individuals is not covered by Ontario healthcare insurance, as evidence is inclusive to whether it improves health outcomes. PSA testing has produced a high rate of false positives and is seen as the “most controversial of all the cancer tests”[[6]](#footnote-7). PSA testing is not a population-based screening approach and therefore is not part of CCO’s Prevention and Screening Program. Currently, CCO has not assigned a department to oversee prostate cancer diagnosis or prostate cancer in general. In September 2008, a Program of Evidenced-Based Care (PEBC) report was issued including surgical and pathological guidelines for radical prostatectomy procedures in prostate cancer management[[7]](#footnote-8). A prostate champion workshop was held a year later in 2009 to assist with guideline implementation and drive the quality improvement agenda forward. Regional surgery, pathology and radiation oncology champions were recruited to assist with implementation, develop the community of practice (CoP) and facilitate continuous improvement in prostate cancer surgery and pathology. In 2011, prostate cancer was identified as a priority for the Disease Pathway Management team to investigate quality improvement areas. In 2012, the Risk Stratification Initiative (RSI) was established with the goal of devising a more accurate mechanism for risk stratification with newly diagnosed prostate cancer cases. A meeting in August 2012 highlighted information gaps in pathology reports. As a result, it was proposed that a mandatory risk stratification field should be added on biopsy forms to ensure that risk data is available in the Ontario Cancer Registry. In October 2012, a CoP Active Surveillance event was held to discuss the need for the development and roll-out of guidelines within their LHIN to diminish variability of practice and improve patient care.

Pancreatic Cancer Treatment

Pancreatic cancer is one of the most deadly cancers with an overall five-year survival rate of 5%; an outcome that has not appreciably changed since the 1970s[[8]](#footnote-9). The vast majority of pancreatic cancer cases are diagnosed after the cancer has spread to regional lymph nodes or more distal metastases. The high mortality rates of pancreatic cancer are also due to poor responses to advanced treatments with little benefit seen from new therapeutics and targeted strategies. Research has been focused on identifying the molecular mechanism behind the disease to identify opportunities for developing strategies for prevention, treatment and early detection. In 2006, PEBC recommended regionalization of hepatic, pancreatic, and biliary Tract (HPB) surgery, detailing the optimum organization for the delivery of surgery in Ontario, including hospital criteria, surgeon criteria and outcome measures. In November 2007, a HPB CoP workshop was held to discuss quality and access issues in response to concerns about increasing volumes and wait times for HPB surgery. As a result, a CoP for HPB surgery was established. The Surgical Oncology Program (SOP) would work with this community to develop and implement initiatives to improve the quality of, and access to HPB surgery in Ontario. In 2011, the original PEBC recommendations of regionalization of HPB surgery were updated.

1. <https://www.cancercare.on.ca/common/pages/UserFile.aspx?fileId=13716> [↑](#footnote-ref-2)
2. <https://www.cancercare.on.ca/common/pages/UserFile.aspx?fileId=13608> [↑](#footnote-ref-3)
3. <https://www.cancercare.on.ca/common/pages/UserFile.aspx?fileId=33455> [↑](#footnote-ref-4)
4. <https://www.cancercare.on.ca/common/pages/UserFile.aspx?fileId=75573> [↑](#footnote-ref-5)
5. <https://www.cancercare.on.ca/common/pages/UserFile.aspx?fileId=125432> [↑](#footnote-ref-6)
6. <https://www.cancercare.on.ca/common/pages/UserFile.aspx?fileId=44610> [↑](#footnote-ref-7)
7. <https://www.cancercare.on.ca/common/pages/UserFile.aspx?fileId=34414> [↑](#footnote-ref-8)
8. <http://icgc.org/icgc/cgp/68/392/810> [↑](#footnote-ref-9)
